# External validation of a web- and artificial intelligence-based HIV/STI risk assessment tool: performance evaluation using data from Sydney sexual health centre

**DOI:** 10.1186/s12879-025-12087-8

**Published:** 2025-11-25

**Authors:** Phyu Mon Latt, Anik Ray, Heng Lu, Nyi N. Soe, Xianglong Xu, Yining Bao, Jason J. Ong, Eric P. F. Chow, Rick Varma, Lei Zhang, Christopher K. Fairley

**Affiliations:** 1 Artificial Intelligence and Modelling in Epidemiology Program, Melbourne Sexual Health Centre, Alfred Health, Melbourne, Australia; 2 School of Translational Medicine, Faculty of Medicine, Nursing and Health Sciences, Monash University, Melbourne, Australia; 3 Sydney Sexual Health Centre, South Eastern Sydney Local Health District, Sydney, NSW 2000 Australia; 4 School of Public Health, Shanghai University of Traditional Chinese Medicine, Shanghai, China; 5 China-Australia Joint Research Centre for Infectious Diseases, School of Public Health, Xi’an Jiaotong University Health Science Centre, Xi’an, Shaanxi Province People’s Republic of China; 6 Melbourne Sexual Health Centre, Alfred Health, Melbourne, Australia; 7 Department of Clinical Research, London School of Hygiene and Tropical Medicine, London, UK; 8 Centre for Epidemiology and Biostatistics, Melbourne School of Population and Global Health, The University of Melbourne, Melbourne, Australia; 9 The Kirby Institute, University of New South Wales, Sydney, Australia; 10 Phase I Clinical Trial Research Ward, The Second Affiliated Hospital of Xi’an Jiaotong University, No. 157 Xi Wu Road, Xi’an, Shaanxi Province 710004 People’s Republic of China

**Keywords:** HIV, Sexually transmitted infections, Artificial intelligence, Risk assessment, Machine learning, External validation, Predictive modelling, Sexual health, Digital health

## Abstract

**Introduction:**

HIV and sexually transmitted infections (STIs) continue to pose significant public health challenges globally. *MySTIRisk*, developed at Melbourne Sexual Health Centre (MSHC), is a machine learning-based tool that predicts individual risk for HIV, syphilis, gonorrhoea, and chlamydia using demographic and behavioural data. While initial validation showed promising results, external validation is crucial to assess its generalisability. This study externally validates *MySTIRisk* using data from the Sydney Sexual Health Centre (SSHC), Australia’s second-largest sexual health centre.

**Methods:**

Following TRIPOD guidelines, we analysed consultations from patients aged 18 years and older attending SSHC between January 2013 and December 2023. Pre-trained *MySTIRisk* models were applied directly without modification. Performance was evaluated using the area under the receiver operating characteristic curve (AUC), sensitivity, and specificity at multiple thresholds, with subgroup analyses across demographic characteristics.

**Results:**

We analysed 159,043 to 207,582 consultations at SSHC, depending on the specific infections tested. The median age was 30 years, and 60.2% to 68.8% of the consultations involved men who have sex with men. The area under the receiver operating characteristic curve (AUC) values using data from SSHC were 0.67 (95% CI: 0.65–0.68) for HIV, 0.70 (95% CI: 0.69–0.71) for syphilis, 0.73 (95% CI: 0.73–0.74) for gonorrhoea, and 0.65 (95% CI: 0.65–0.66) for chlamydia, which were lower than the original MSHC validation metrics (0.74–0.87, all *p* < 0.001). Notably, model performance varied across demographic subgroups, with stronger HIV prediction among men who have sex with men with an AUC of 0.78 and better gonorrhoea prediction among younger attendees < 25 years with an AUC of 0.79. At balanced sensitivity-specificity thresholds, the models identified 58.6–64.1% of infections while requiring testing of only 25.8–39.4% of the population.

**Conclusions:**

Despite performance decrements in external validation using SSHC data, *MySTIRisk* maintained moderate to good predictive ability across all infections, demonstrating reasonable generalisability across different clinical populations. The demographic variations in performance highlight the importance of context-specific implementation and potential recalibration to optimise clinical utility.

**Clinical trial number:**

Not applicable.

**Supplementary Information:**

The online version contains supplementary material available at 10.1186/s12879-025-12087-8.

## Introduction

Sexually transmitted infections (STIs) and HIV pose a significant public health challenge, with growing infection rates despite prevention efforts [1–3]. Early detection is essential to reducing transmission and complications, yet many individuals delay testing due to limited awareness, barriers in healthcare access, social stigma and the frequently asymptomatic presentation of the infection [4–6].

Risk assessment tools can help optimise resource use in busy clinical settings, where universal testing may not be feasible due to constraints in staffing, supplies, or funding [7, 8]. By identifying individuals most likely to have infections, these tools can support earlier diagnosis while avoiding unnecessary testing in low-risk groups, thereby improving overall testing efficiency [9]. Existing risk prediction tools primarily focus on HIV and rely on conventional statistical methods, such as logistic regression [10, 11]. There remains an unmet need for tools that assess multiple STIs simultaneously while integrating more sophisticated analytical approaches. Machine learning offers potential benefits in this context, including the ability to handle complex, nonlinear, and multidimensional associations between risk factors and outcomes [12, 13].

To address this gap, the Melbourne Sexual Health Centre (MSHC) recently developed *MySTIRisk*, a machine learning-based tool for predicting individual risk of HIV, syphilis, gonorrhoea, and chlamydia [9, 14]. *MySTIRisk* uses demographic and behavioural data from the clinic attendees to generate personalised risk scores. Initial testing at MSHC showed promising results, with the area under the curve (AUC) values ranging from 0.70 to 0.84 across the four infections, indicating good discriminative ability [9].

However, *MySTIRisk* was developed and validated using data from a single centre. To ensure its generalisability and wider applicability, external validation is necessary. Previous research has shown that machine learning models often exhibit performance variability when applied to new populations due to demographic and behavioural differences [15–17]. Our recent systematic review of machine learning-based STI risk prediction tools revealed that only a small proportion of studies conducted external validation, with most limited to temporal validation within the same population [18]. Without external validation, the effectiveness and reliability of *MySTIRisk* across diverse populations remain uncertain. This is particularly important for machine learning models, which can be sensitive to the specific characteristics of the training data [19, 20]. External validation helps assess whether the tool can be applied more broadly across Australian sexual health settings or requires adjustments for local implementation.

This study aims to conduct the external validation of *MySTIRisk* using data from the Sydney Sexual Health Centre (SSHC), assessing *MySTIRisk’s* predictive performance for all four STIs among the attendees at the SSHC.

## Methods

### Study population and design

We conducted an external validation study of the *MySTIRisk* tool using data from SSHC, following the Transparent Reporting of a Multivariable Prediction Model for Individual Prognosis or Diagnosis (TRIPOD) statement guidelines for Type 2b studies (external validation without model updating) [21]. This included clearly defining the study population, eligibility criteria, predictor variables, outcome measures, statistical methods, and performance metrics consistent with TRIPOD checklist items. We used clinical consultation data from patients aged 18 years or older who attended SSHC between January 2013 and December 2023. Transgender individuals were excluded to maintain consistency with the original model development study, as the initial dataset did not include sufficient representation of this group.

We selected SSHC for validation because it is the second largest sexual health centre in Australia, serving a diverse patient population with different demographics than MSHC. Together, MSHC and SSHC provide sexual health services to a substantial proportion of Australia’s urban population, attending a free, publicly funded service. A detailed cross-site comparison is provided in Supplementary Tables S1a–S1d.

### Data collection and cleaning

At SSHC, we extracted data retrospectively from the electronic health record (EHR) of registered attendees, including patient demographics, sexual behaviours, recent STI contact and previous STI diagnoses, and HIV/STI test results.

We performed systematic data cleaning to ensure compatibility with the original *MySTIRisk* model development approach. This included handling missing values, standardising categorical variables, and converting continuous variables to the appropriate format. Missing data were not imputed but treated as a separate category, consistent with the approach used in the original model development [12, 14]. We organised the data into four separate datasets corresponding to each infection endpoint (HIV, syphilis, gonorrhoea, and chlamydia). Testing decisions were based on clinical assessment, patient risk factors, symptoms, and evolving Australian guidelines over the study period. Each dataset included consultations where the respective infection was tested.

### Outcome definitions

We used the same outcome definitions as the original *MySTIRisk* development study [12, 14]. HIV infection was defined as a new diagnosis of HIV based on serology without confirmatory testing, consistent with the original model development approach. Syphilis infection was defined as a new diagnosis of early syphilis (primary, secondary, or early latent using serological testing or nucleic acid amplification test (NAAT)). Gonorrhoea infection was defined as a new diagnosis using culture or NAAT at any anatomical site. Chlamydia infection was defined as a new diagnosis using NAAT at any anatomical site. All diagnoses were coded by clinicians following established clinical guidelines and standard laboratory procedures.

### Predictor variables

We used the same predictor variables identified in the original *MySTIRisk* tool development study [12, 14]. While other factors, such as partner notification, may influence testing decisions, we maintained consistency with the original model variables to enable valid comparisons. Key predictors were listed in the next section. However, the “sex overseas” variable was not available in the SSHC dataset and was coded as missing for all records.

For categorical variables, we used the same category definitions as the original study. For instance, condom use was categorised as “always,” “never,” “sometimes,” or “not applicable”, ensuring consistency with prior classifications. For numerical variables such as age and number of sexual partners, we maintained the same scaling and transformation approaches used in the original models.

### Risk assessment models

*MySTIRisk* is a web-based tool that collects demographic and behavioural information through a questionnaire, including age, number of casual sexual partners (male and female) in the last 12 months, condom use with partners in the previous 12 months, sex overseas in the last 12 months, injecting drug use in the last 12 months, recent STI contact and previous STI diagnoses, and presence of STI symptoms. The tool applies machine learning algorithms to generate personalised risk scores for each of the four infections. The complete questionnaire and interface are publicly available at the *MySTIRisk* website [22], and a detailed development methodology is provided in the original studies [9, 14].

In brief, the *MySTIRisk* comprises different machine learning models for each infection type. For HIV prediction, an ensemble approach combining elastic net regression, gradient boosting machine (GBM), and random forest models was used. For syphilis prediction, a GBM model was employed. Gonorrhoea risk was predicted using a random forest model, while chlamydia prediction utilised a GBM model [9]. Full details of these model developments can be found in previously published studies [9, 12, 14]. In brief, the development process used 5-fold cross-validation with data from the MSHC (2015–2018), with 80% used for training and 20% for validation.

For our external validation, we applied these pre-trained models directly to the SSHC dataset without retraining or adjustments, following established external validation methodology [23]. This approach ensures a robust assessment of model generalisability in a different clinical population without introducing modifications that might bias results.

### Statistical analysis

We calculated the area under the receiver operating characteristic curve (AUC) with bootstrapped 95% confidence intervals derived using 1,000 resamples to evaluate the model’s discrimination performance for each infection. The AUC measures a model’s ability to distinguish between positive and negative cases, with values ranging from 0.5 (no discriminative ability, equivalent to random chance) to 1.0 (perfect discrimination) [24]. The bootstrapping process provided robust estimates of variability in AUC values. We then compared these results with the performance metrics from the original *MySTIRisk* validation dataset (20% testing data from MSHC).

To visually compare model performance, we overlaid receiver operating characteristic (ROC) curves from MSHC and SSHC validation datasets for each infection onto a single chart. We performed a Z-test to analyse differences between AUC values derived from the two independent datasets. This test relied on bootstrapped AUC distributions to calculate the Z-statistic and determine whether the differences were statistically significant. We considered a p-value of < 0.05 to indicate statistical significance.

To evaluate the model’s performance across clinically relevant risk thresholds, we calculated sensitivity and specificity at three key cutoffs for each infection: high sensitivity (targeting 90.0%), balanced sensitivity and specificity (determined using Youden’s index), and high specificity (targeting 90.0%). Youden’s index identifies the optimal threshold that maximises both sensitivity and specificity simultaneously, calculated as J = sensitivity + specificity − 1, with values closer to 1 indicating better performance [25, 26]. For each threshold, we also computed positive predictive value (PPV), negative predictive value (NPV), and the percentage of the population requiring testing. To calculate confidence intervals, we used the Wilson score method to estimate confidence intervals for proportion-based metrics in binary classification [27].

Additionally, we conducted subgroup analyses to assess model performance consistency across key demographic characteristics. We calculated AUC values with 95% confidence intervals for population subgroups defined by population groups, age, and country of birth.

For calibration assessment, we evaluated how well predicted probabilities aligned with observed outcomes. Following the original study’s methodology, we divided predictions into 200 equally sized subgroups sorted by predicted probability and calculated the observed prevalence within each subgroup. We fitted logistic functions to these data points to create smooth calibration curves and visualise the relationship between predicted probabilities and observed prevalence. All statistical analyses were conducted using the Python programming language (version 3.9.12).

## Results

### Demographic, behavioural, and clinical characteristics

Between January 2013 and December 2023, we analysed 159,043 consultations for HIV, 168,443 for syphilis, and 207,582 for both gonorrhoea and chlamydia at the SSHC. Table 1 summarises the demographic and behavioural characteristics of these consultations. The median age was approximately 30 years across all datasets, with men who have sex with men (MSM) representing most of the consultations (60.2–68.8%). Most attendees (64.2–65.5%) were born overseas, and inconsistent condom use was reported in nearly half of all consultations.

**Table 1:** Characteristics of clinic consultations in individual data sets

Predictors	HIV (*N* = 159043 Consultations)	Syphilis (*N* = 168443 Consultations)	Gonorrhoea (*N* = 207582 Consultations)	Chlamydia (*N* = 207582 Consultations)
**Age**,** median (IQR)**	30 (25–37)	30 (25–37)	29 (25–36)	29 (25–36)
**Country of birth**,** n (%)**				
Australia and New Zealand	56,949 (35.8)	60,336 (35.8)	71,583 (34.5)	71,583 (34.5)
Overseas	102,094 (64.2)	108,107 (64.2)	135,999 (65.5)	135,999 (65.5)
**STI symptoms**,** n (%)**				
Present	29,953 (18.9)	32,115 (19.1)	50,575 (24.4)	50,496 (24.3)
Absent	129,090 (81.2)	136,328 (81.0)	157,007 (75.6)	157,086 (75.7)
**Population type**,** n (%)**				
MSM	109,362 (68.8)	115,933 (68.8)	124,963 (60.2)	124,964 (60.2)
Heterosexual male	23,102 (14.5)	25,516 (15.2)	37,229 (18.0)	37,228 (17.9)
Female	26,579 (16.7)	26,994 (16.0)	45,390 (21.9)	45,390 (21.9)
**Condom use with male partners**,** n (%)**				
Always	41,447 (26.1)	43,463 (25.8)	48,936 (23.6)	48,935 (23.6)
Sometimes	76,499 (48.1)	79,712 (47.3)	100,084 (48.2)	100,095 (48.2)
Never	27,167 (17.1)	29,221 (17.4)	38,796 (18.7)	38,795 (18.7)
Not Applicable	10,330 (6.5)	11,510 (6.8)	13,240 (6.4)	13,235 (6.4)
Unsure/Decline	46 (0.0)	50 (0.0)	74 (0.0)	74 (0.0)
Missing	3554 (2.2)	4487 (2.7)	6452 (3.1)	6448 (3.1)
Number of male sexual partners in last 12 months, median (IQR)	7 (3–15)	7 (3–15)	6 (3–15)	6 (3–15)
Number of female sexual partners in last 12 months, median (IQR)	3 (1–7)	3 (1–6)	3 (1–7)	3 (1–7)
**Last time injected drugs not prescribed by doctor**,** n (%)**				
Never	156,433 (98.4)	165,357 (98.2)	204,079 (98.3)	204,077 (98.3)
Less than 3 months	1396 (0.9)	1720 (1.0)	1963 (1.0)	1962 (1.0)
3–12 months	577 (0.4)	709 (0.4)	789 (0.4)	789 (0.4)
More than 12 months	637 (0.4)	657 (0.4)	751 (0.4)	754 (0.4)
**Past history of gonorrhoea**,** n (%)**				
Yes	44,512 (28.0)	49,445 (29.4)	61,432 (29.6)	61,420 (29.6)
No	114,516 (72.0)	118,982 (70.6)	146,131 (70.4)	146,143 (70.4)
Missing	15 (0.0)	16 (0.0)	19 (0.0)	19 (0.0)
**Past history of nonspecific urethritis**,** n (%)**				
Yes	16,453 (10.4)	17,972 (10.7)	24,232 (11.7)	24,211 (11.7)
No	142,575 (89.6)	150,455 (89.3)	183,331 (88.3)	183,352 (88.3)
Missing	15 (0.0)	16 (0.0)	19 (0.0)	19 (0.0)
**Past history of syphilis**,** n (%)**				
Yes	19,757 (12.4)	23,550 (14.0)	28,580 (13.8)	28,557 (13.8)
No	139,271 (87.6)	144,877 (86.0)	178,983 (86.2)	179,006 (86.2)
Missing	15 (0.0)	16 (0.0)	19 (0.0)	19 (0.0)
**Sexual contact with someone diagnosed with gonorrhoea**,** n (%)**				
Yes	3386 (2.1)	3635 (2.1)	5409 (2.6)	5381 (2.6)
No	155,657 (97.9)	164,808 (97.9)	202,173 (97.4)	202,201 (97.4)
**Sexual contact with someone diagnosed with chlamydia**,** n (%)**				
Yes	4657 (2.9)	4870 (2.9)	8578 (4.1)	8587 (4.1)
No	154,386 (97.1)	163,573 (97.1)	199,004 (95.9)	198,995 (95.9)
**Sexual contact with someone diagnosed with syphilis**,** n (%)**				
Yes	1426 (0.9)	1848 (1.1)	1573 (0.8)	1572 (0.7)
No	157,617 (99.1)	166,595 (98.9)	206,009 (99.2)	206,010 (99.3)
**Infection positivity**,** n (%)**				
Positive	1163 (0.7)	3410 (2.0)	15,423 (7.4)	20,801 (10.0)
Negative	157,880 (99.3)	165,033 (98.0)	192,159 (92.6)	186,781 (90.0)

The HIV/STI positivity at SSHC were 0.7% for HIV, 2.0% for syphilis, 7.4% for gonorrhoea, and 10.0% for chlamydia (Table 1). Comparatively, MSHC reported lower positivity, with 0.3% for HIV, 1.7% for syphilis, 5.9% for gonorrhoea, and 8.1% for chlamydia [9], indicating potential variations in patient demographics, testing patterns, or underlying transmission dynamics between the two centres.

### External validation performance

**Fig. 1:**
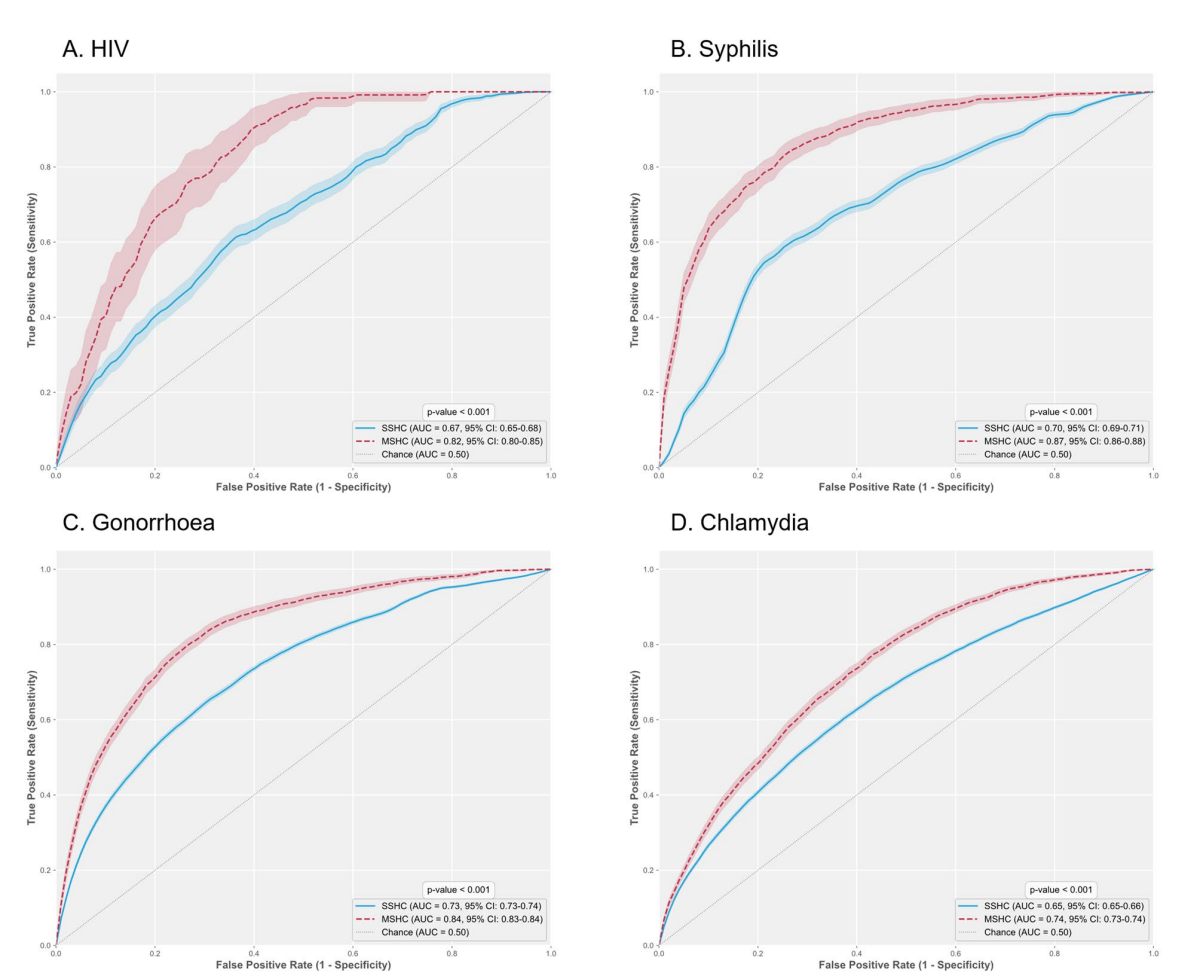
External validation of *MySTIRisk*: model performance across two sexual health centres

The AUC values for SSHC were 0.67 (95% CI: 0.65–0.68) for HIV, 0.70 (95% CI: 0.69–0.71) for syphilis, 0.73 (95% CI: 0.73–0.74) for gonorrhoea, and 0.65 (95% CI: 0.65–0.66) for chlamydia. These results were significantly lower than the performance metrics at MSHC, which showed AUC values of 0.82 (95% CI: 0.80–0.85) for HIV, 0.87 (95% CI: 0.86–0.88) for syphilis, 0.84 (95% CI: 0.83–0.84) for gonorrhoea, and 0.74 (95% CI: 0.73–0.74) for chlamydia. Statistical comparison revealed significant differences in predictive performance across all four infections (all p-values < 0.001). Figure 1 displays the ROC curves for both centres across all four infections, illustrating consistent differences in model discrimination between these distinct clinical populations.

### Subgroup analysis

*MySTIRisk* demonstrated varying performance across demographic subgroups (Table 2). The HIV model showed better discrimination among MSM (AUC 0.78, 95% CI: 0.74–0.81) compared to heterosexual males (AUC 0.65, 95% CI: 0.63–0.68, *p* = 0.06) and females (AUC 0.61, 95% CI: 0.59–0.64, *p* = 0.002). For gonorrhoea, performance was markedly lower in females (AUC 0.57, 95% CI: 0.55–0.59, *p* = 0.2) compared to MSM. Age-related variations were most notable for gonorrhoea, where younger attendees (< 25 years) exhibited significantly higher predictive performance (AUC 0.79, 95% CI: 0.78–0.80) than other age groups. The chlamydia model showed the most consistent performance across demographic categories (AUCs 0.64–0.67). Country of birth had minimal impact on model performance for all infections. Subgroup analysis by symptom presence showed better performance among symptomatic patients (AUC 0.67–0.84) than asymptomatic patients (AUC 0.62–0.69), though this likely reflects symptoms being a key model predictor.

**Table 2:** Subgroup analyses of *mystirisk* model performance across key demographics

Subgroup	HIV	Syphilis	Gonorrhoea	Chlamydia
**Overall**	**0.67 (0.65–0.68)**	**0.70 (0.69–0.71)**	**0.73 (0.73–0.74)**	**0.65 (0.65–0.66)**
Population type				
MSM (reference)	0.78 (0.74–0.81)	0.70 (0.68–0.71)	0.71 (0.70–0.71)	0.66 (0.65–0.66)
Heterosexual male	0.65 (0.63–0.68)	0.63 (0.61–0.66)***	0.72 (0.71–0.73)	0.66 (0.65–0.67)
Female	0.61 (0.59–0.64)**	0.67 (0.62–0.71)	0.57 (0.55–0.59)***	0.64 (0.63–0.65)**
Age				
< 25 years	0.64 (0.62–0.66)***	0.73 (0.70–0.75)***	0.79 (0.78–0.80)***	0.66 (0.65–0.67)
25–34 years (reference)	0.68 (0.64–0.72)	0.68 (0.66–0.69)	0.72 (0.72–0.73)	0.65 (0.65–0.66)
≥ 35 years	0.74 (0.68–0.80)*	0.70 (0.69–0.72)*	0.70 (0.69–0.70)***	0.64 (0.63–0.65)
Country of birth				
Australia and New Zealand (reference)	0.65 (0.62–0.69)	0.71 (0.69–0.72)	0.72 (0.71–0.73)	0.67 (0.66–0.67)
Overseas	0.66 (0.65–0.68)	0.70 (0.69–0.71)	0.74 (0.73–0.74)***	0.65 (0.64–0.65)***
Presence of symptoms^#^				
Yes (reference)	0.84 (0.82–0.86)	0.71 (0.70–0.72)	0.79 (0.78–0.79)	0.67 (0.66–0.67)
No	0.62 (0.61–0.64)***	0.66 (0.65–0.67)***	0.69 (0.69–0.70)***	0.64 (0.64–0.65)***

*p < 0.05, **p < 0.01, ***p < 0.001 compared to reference group

MSM: men who have sex with men; AUC: area under the receiver operating characteristic curve; CI: confidence interval

^#^STI symptoms refer to self-reported symptoms commonly associated with sexually transmitted infections, such as genital discharge, ulcers, and pain during urination or sex

### Threshold analysis

Table 3 presents the performance of *MySTIRisk* across multiple risk thresholds for each infection. At thresholds calibrated for high sensitivity (90.0%), the proportion of the population requiring testing ranged from 70.3% (gonorrhoea) to 81.3% (chlamydia), with corresponding specificities between 19.7% and 31.3%. When optimising for balanced sensitivity and specificity, the models achieved more moderate but clinically useful performance. For HIV, a threshold of 0.62 resulted in 61.0% sensitivity (95% CI: 58.2–63.8%) and 64.1% specificity (95% CI: 63.8–64.3%), requiring testing of only 36.1% of the population. For syphilis, gonorrhoea, and chlamydia, the balanced thresholds achieved sensitivities of 58.6%, 64.1%, and 60.1%, respectively, with specificities ranging from 62.9% to 74.9%. At high specificity thresholds (90.0%), the models demonstrated lower sensitivities (23.5–37.0%) but substantially reduced the testing proportion to 10.1–12.0% of the population.

**Table 3:** Performance of the *mystirisk* models at different risk thresholds

Infections	Scenario	Threshold	Sensitivity (95% CI)	Specificity (95% CI)	PPV (95% CI)	NPV (95% CI)	% Population Tested
HIV	Sensitivity at 90%	0.36	89.9% (88.1–91.5%)	27.2% (27.0-27.5%)	0.9% (0.8-1.0%)	99.7% (99.7–99.8%)	72.9%
	Balanced Sensitivity and Specificity*	0.62	61.0% (58.2–63.8%)	64.1% (63.8–64.3%)	1.2% (1.1–1.3%)	99.6% (99.5–99.6%)	36.1%
	Specificity at 90%	0.68	26.0% (23.5–28.6%)	90.0% (89.9–90.1%)	1.9% (1.7–2.1%)	99.4% (99.4–99.4%)	10.1%
Syphilis	Sensitivity at 90%	0.25	90.1% (89.0–91.0%)	25.6% (25.4–25.9%)	2.4% (2.4–2.5%)	99.2% (99.1–99.3%)	74.7%
	Balanced Sensitivity and Specificity*	0.46	58.6% (56.9–60.2%)	74.9% (74.7–75.1%)	4.6% (4.4–4.8%)	98.9% (98.8–98.9%)	25.8%
	Specificity at 90%	0.72	23.5% (22.1–25.0%)	90.1% (89.9–90.2%)	4.7% (4.4-5.0%)	98.3% (98.2–98.3%)	10.2%
Gonorrhoea	Sensitivity at 90%	0.33	90.0% (89.5–90.5%)	31.3% (31.1–31.5%)	9.5% (9.4–9.7%)	97.5% (97.4–97.6%)	70.3%
	Balanced Sensitivity and Specificity*	0.57	64.1% (63.3–64.9%)	70.3% (70.1–70.5%)	14.8% (14.5–15.0%)	96.1% (96.0-96.2%)	32.3%
	Specificity at 90%	0.66	37.0% (36.2–37.7%)	90.0% (89.9–90.1%)	22.9% (22.4–23.4%)	94.7% (94.6–94.8%)	12.0%
Chlamydia	Sensitivity at 90%	0.37	90.0% (89.6–90.4%)	19.7% (19.5–19.9%)	11.1% (10.9–11.2%)	94.6% (94.4–94.9%)	81.3%
	Balanced Sensitivity and Specificity*	0.52	60.1% (59.4–60.7%)	62.9% (62.7–63.1%)	15.3% (15.0-15.5%)	93.4% (93.3–93.5%)	39.4%
	Specificity at 90%	0.63	26.6% (26.0-27.2%)	90.0% (89.9–90.1%)	22.8% (22.3–23.4%)	91.7% (91.5–91.8%)	11.7%

Sensitivity represents the percentage of true positive cases correctly identified by the model, while specificity measures the percentage of true negative cases correctly classified. The positive predictive value (PPV) represents the proportion of positive results that are truly positive cases, whereas the negative predictive value (NPV) reflects the proportion of negative results that are truly negative cases. The percentage of the population tested corresponds to the proportion of individuals who exceeded the given probability threshold

### Calibration assessment

**Fig. 2:**
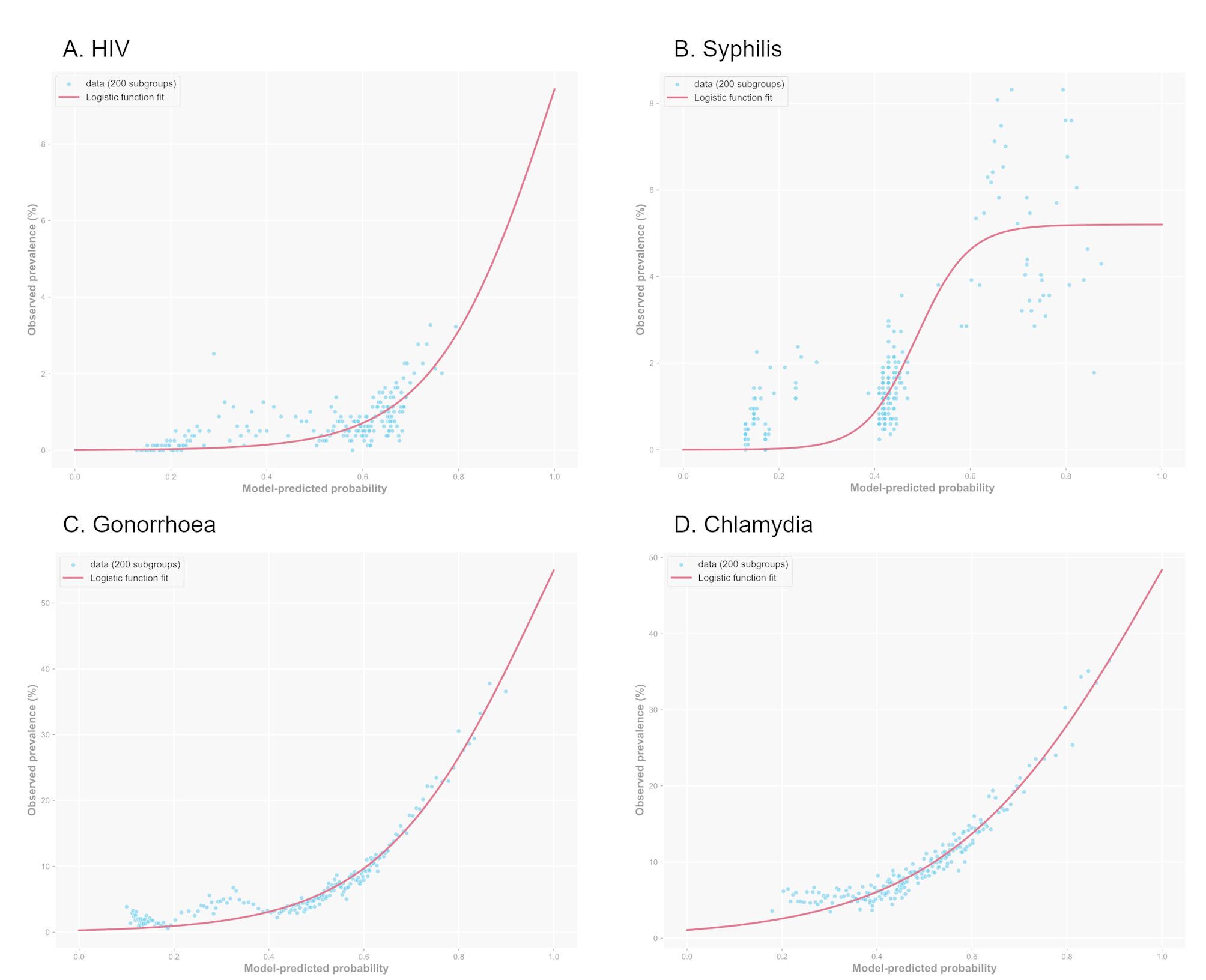
Calibration plots of model-predicted probabilities versus observed prevalence for *MySTIRisk* models on SSHC data

In our calibration assessment, we examined the association between predicted probabilities and observed prevalence for all four STIs (Fig. 2), as mentioned in the development study of *MySTIRisk* [14]. The calibration plots demonstrated excellent agreement between predicted and observed risk across the probability spectrum for gonorrhoea and chlamydia, with data points closely following the logistic function fit. For HIV, the model showed good calibration at lower risk probabilities but slightly underestimated infection rates at higher probabilities (>0.7), although this region contained fewer data points. The syphilis model demonstrated the most variation in calibration, with greater scatter around the fitted curve, particularly at mid-range probabilities (0.4–0.7).

## Discussion

Our external validation of *MySTIRisk* demonstrated moderate to good predictive performance for all four STIs using the data from SSHC, although with significant decrements compared to the original *MySTIRisk* models developed at the MSHC. We found AUC values ranging from 0.65 for chlamydia and 0.73 for gonorrhoea, reflecting reasonable discriminative ability across infections, though lower than the original MSHC values (0.74–0.87). This performance decline aligns with established patterns in machine learning validation studies, where models typically show reduced effectiveness when applied to external populations due to differences in demographics, behavioural patterns, and testing practices [15, 16, 28]. Key differences between sites included SSHC’s higher proportion of overseas-born attendees, greater MSM representation, and paradoxically higher infection rates despite fewer symptomatic presentations. We observed notable population-specific variations, with the models performing better for HIV prediction among MSM (AUC 0.78) and for gonorrhoea among younger attendees (AUC 0.79). These findings underscore the importance of context-specific considerations in AI-based risk assessment tools. While *MySTIRisk* retains clinical utility across diverse settings, our results highlight the necessity of local validation and potential recalibration before widespread implementation, consistent with recent studies on clinical prediction models in infectious disease settings [9, 13, 15–17].

Our study demonstrated that *MySTIRisk* models exhibited consistently lower discriminative performance when using the data from SSHC, with AUC values at 0.65–0.73 markedly reduced from those reported at MSHC (0.74–0.87). While such performance decrements are anticipated in external validation studies of machine learning models, several specific factors may explain these observed differences. First, the demographic and epidemiological composition differed substantially between sites. The SSHC dataset comprised a higher proportion of overseas-born attendees (approximately 65%) and MSM and lower proportions of heterosexual individuals than the more evenly distributed MSHC population. These population differences may influence how risk factors manifest and interact across settings. Second, differences in clinical documentation protocols may have affected model input quality. For example, while MSHC recorded sexual encounters overseas (“sex with someone outside Australia or New Zealand or had sex in Australia with someone from overseas”), SSHC did not capture this risk factor. Third, despite having fewer symptomatic presentations (18.8–24.4% vs. 28.3–35.7% at MSHC), SSHC showed higher positivity across all infections, indicating population-specific testing and risk dynamics that models may struggle to capture. Studies have demonstrated that such disparities significantly impact machine learning model performance during external validation, particularly for models developed using comprehensive datasets [29, 30]. Fourth, the methods of triage, testing protocols, and patterns of symptomatic individuals may vary between the two populations. These differences may have led to differences in the patterns of risk factors for each infection between the MSHC and SSHC populations. Despite these differences, it is encouraging that *MySTIRisk* maintained moderate discriminative ability using the SSHC data. This suggests that the core risk factors identified during development retain predictive value in at least one other major Australian sexual health centre.

Our subgroup analyses revealed important differences in *MySTIRisk*’s performance across demographic categories. For HIV prediction, the markedly superior performance among MSM (AUC 0.78) compared to heterosexual populations (AUC 0.61–0.65) suggested that the model captured MSM-specific risk patterns more effectively. This likely reflects more uniform risk profiles in this population, where transmission dynamics and behavioural predictors have been more thoroughly documented. Age-stratified analysis demonstrated that younger attendees (< 25 years) showed significantly higher prediction accuracy for gonorrhoea (AUC 0.79) compared to other age groups. While Australian guidelines recommend opportunistic STI screening for younger populations [31], the mechanisms underlying this improved model performance remain unclear and warrant further investigation. Interestingly, country of birth had minimal impact on model performance except for gonorrhoea and chlamydia. These demographic disparities in predictive performance align with findings from Franklin et al. [32], which demonstrated that demographic characteristics can introduce bias in machine learning models, affecting their generalisability across diverse populations. The observed variations in predictive performance across subgroups indicate that risk factors have different predictive weights in diverse populations, reflecting the complex interplay of behavioural, social, and biological determinants of STI transmissions [33, 34].

The threshold analysis findings provided valuable guidance for implementing *MySTIRisk* in diverse clinical settings. Setting risk thresholds to achieve high sensitivity (90.0%) would capture most infections while reducing testing volume compared to universal testing. Specifically, 70.3–81.3% of attendees would be tested under this approach, representing a modest reduction of 18.7–29.7%. However, this level of testing may still place a considerable strain on resources, particularly in high-volume clinics, and may not offer substantial efficiency gains relative to the simplicity of universal testing. Conversely, high specificity thresholds (90.0%) would significantly reduce testing volume to only 10.1–12.0% of the population but would result in missing 63.0–76.5% of infections, potentially limiting early detection efforts. A balanced approach using Youden’s index thresholds could represent an optimal middle ground by identifying 58.6–64.1% of infections while reducing the testing proportion to 25.8–39.4%. However, threshold selection should consider infection-specific priorities. Missing HIV or syphilis cases carries severe clinical and public health consequences, so high sensitivity is essential. For gonorrhoea, antimicrobial stewardship may justify higher specificity, while for chlamydia, a balanced or sensitivity-focused threshold may be appropriate, given the risk of complications in young women [35]. In resource-limited settings, *MySTIRisk* could help prioritise testing for individuals at higher risk. Meanwhile, in well-resourced environments, it could complement universal testing by identifying candidates for more comprehensive screening. The flexibility to adjust thresholds based on specific clinical contexts represents a key advantage of machine learning-based risk assessment tools compared to traditional screening questionnaires, which rely on fixed criteria and may lack adaptability to evolving epidemiological trends [14].

Future research should address several key areas to enhance the clinical utility of *MySTIRisk* and similar AI-based prediction tools. First, transfer learning approaches could be explored to adapt models to specific clinical settings while preserving their core predictive capabilities. This would involve fine-tuning the existing models with local data rather than complete retraining, potentially improving performance while maintaining generalisability [36]. Second, future iterations should expand demographic representation, particularly including trans and gender diverse individuals who were excluded from current analyses, as there were no specific sexual behavioural questions for this population at the time when *MySTIRisk* was developed. Third, prospective implementation studies are needed to evaluate the real-world impact of integrating *MySTIRisk* into clinical workflows, including assessment of resource utilisation, infection detection rates, and user acceptance. Such studies should examine *MySTIRisk’s* specific clinical applications, including risk-based testing triage, patient counselling, and resource allocation strategies. While the tool is publicly available, formal integration into clinical workflows requires assessment of user acceptance and workflow feasibility. Additional research could explore the temporal stability of these predictions, as sexual behaviour patterns and infection dynamics evolve over time [37]. Finally, comparative effectiveness research should evaluate whether AI-based risk stratification outperforms traditional clinician judgment or simpler risk assessment tools in terms of both accuracy and cost-effectiveness. It would strengthen the evidence base for the broader adoption of machine learning approaches in sexual health services and inform best practices for their implementation across diverse healthcare settings.

Our study has several notable strengths. This is the first external validation of an AI-based HIV/STI risk assessment tool across Australia’s two largest publicly funded sexual health centres, representing a significant proportion of the country’s urban sexual health services and providing robust statistical power through its extensive sample size. Our adherence to TRIPOD guidelines for prediction model validation ensures methodological quality and transparent reporting. The comprehensive assessment across four key STIs offers valuable comparative insights into prediction patterns across different infections. Additionally, our analysis of multiple risk thresholds provides practical implementation guidance that can be tailored to various clinical scenarios. By validating *MySTIRisk* in a demographically distinct setting, we have tested the model’s resilience to population heterogeneity, a critical consideration for AI tools intended for widespread implementation across diverse healthcare environments.

Our study has several important limitations that warrant consideration when interpreting the results. First, the retrospective nature of our analyses may have introduced selection bias, though our large sample size spanning a decade helps mitigate some temporal variation effects. Second, we excluded transgender individuals to maintain consistency with the original model development, representing a significant gap in generalisability that future iterations must address. This exclusion was intentional because data systems did not capture epidemiological risk on these individuals until recently. With time, adequate epidemiological risk data will accumulate and allow this analysis in the future. Third, differences in clinical documentation between sites meant that some risk factors used in the original model development were not identically captured at SSHC, potentially affecting model performance. Although we attempted to standardise variables where possible, some differences were unavoidable due to inherent variations in clinical workflows between centres. Fourth, while the study included a diverse patient population, both centres are urban sexual health clinics, potentially limiting generalisability to rural settings or primary care contexts where STI testing also occurs. However, these two centres serve the largest metropolitan areas in Australia, providing substantial population coverage. Finally, we did not assess the models’ performance for predicting multiple concurrent infections, which represents a clinically important scenario, as the original model development focused on individual infections.

In conclusion, our external validation of *MySTIRisk* demonstrated moderate to good predictive performance across all four STIs, though with decreased discrimination compared to the original development site. Despite this performance drop, the tool maintained discriminative ability across different clinical populations, supporting its potential application in diverse sexual health settings. Population-specific variations highlight the need for contextualised implementation, with adjustable thresholds offering flexibility based on local resources and priorities. While improvements are needed, this first external validation across Australia’s two largest sexual health centres provides evidence supporting the broader application of AI-based risk assessment in sexual health, though local validation remains essential before widespread implementation.

## Supplementary Information

Below is the link to the electronic supplementary material.

Supplementary Material 1

## Data Availability

The data supporting this study’s findings are not openly available due to sensitivity and are available from the corresponding author, Dr. Phyu Mon Latt, at phyu.latt@monash.edu, upon reasonable request. Data are in controlled access data storage at the Melbourne Sexual Health Centre.

## Abbreviations

AUC: Area under the curve
CI: Confidence Interval
EHR: Electronic health record
GBM: Gradient boosting machine
IQR: Interquartile Range
MSHC: Melbourne Sexual Health Centre
MSM: Men who have sex with men
NAAT: Nucleic acid amplification test
NHMRC: National Health and Medical Research Council
NPV: Negative predictive value
PPV: Positive predictive value
ROC: Receiver operating characteristic
SESLHD: South Eastern Sydney Local Health District
SSHC: Sydney Sexual Health Centre
STI: Sexually transmitted infections
TRIPOD: Transparent Reporting of a Multivariable Prediction Model for Individual Prognosis or Diagnosis
