# Supplementary material for: External validation of a web- and artificial intelligence-based HIV/STI risk assessment tool: performance evaluation using data from Sydney sexual health centre
Source: BMC Infect Dis. 2025 Nov 25;25:1647. doi: 10.1186/s12879-025-12087-8 (PMC12648915; doi:10.1186/s12879-025-12087-8)
Supplement: Supplementary file 1 — Supplementary Material 1 [file 12879_2025_12087_MOESM1_ESM.docx]

Supplementary Table S1a. Demographic and Clinical Characteristics Comparison: HIV Dataset

| **Predictors** | **MSHC (N = 216252)** | **SSHC (N = 159043)** |
| --- | --- | --- |
| **Age, median (IQR)** | 29 (25–35) | 30 (25-37) |
| **Country of birth, n (%)** |  |  |
| Australia and New Zealand | 102350 (47.3) | 56949 (35.8) |
| Overseas | 104085 (48.1) | 102094 (64.2) |
| Missing |  |  |
| **Population type, n (%)** |  |  |
| MSM | 105616 (48.8) | 109362 (68.8) |
| Heterosexual male | 43716 (20.2) | 23102 (14.5) |
| Female | 66920 (30.9) | 26579 (16.7) |
| **Condom use with male partners, n (%)** | |  |
| Always | 40759 (18.8) | 41447 (26.1) |
| Sometimes | 90136 (41.7) | 76499 (48.1) |
| Never | 16289 (7.5) | 27167 (17.1) |
| Not Applicable | 3752 (1.7) | 10330 (6.5) |
| Unsure/Decline | 7896 (3.7) | 46 (0.0) |
| Missing | 57420 (26.6) | 3554 (2.2) |
| **Last time injected drugs not prescribed by a doctor, n (%)** | |  |
| Never | 204029 (94.3) | 156433 (98.4) |
| Less than 3 months | 2588 (1.2) | 1396 (0.9) |
| 3-12 months | 908 (0.4) | 577 (0.4) |
| More than 12 months | 2453 (1.1) | 637 (0.4) |
| Decline/Unsure | 4669 (2.2) | - |
| Missing | 1605 (0.7) | - |
| **Past history of gonorrhoea, n (%)** |  |  |
| Yes | 35939 (16.6) | 44512 (28.0) |
| No | 51906 (24.0) | 114516 (72.0) |
| Unsure | 3803 (1.8) | - |
| Missing | 124604 (57.6) | 15 (0.0) |
| **Past history of syphilis, n (%)** |  |  |
| Yes | 2412 (1.1) | 19757 (12.4) |
| No | 85433 (39.5) | 139271 (87.6) |
| Unsure | 3803 (1.8) | - |
| Missing | 124604 (57.6) | 15 (0.0) |
| **Infection positivity, n (%)** |  |  |
| Positive | 593 (0.3) | 1163 (0.7) |
| Negative | 215659 (99.7) | 157880 (99.3) |

MSHC: Melbourne Sexual Health Centre; SSHC: Sydney Sexual Health Centre; MSM: men who have sex with men; IQR: interquartile range

Statistical comparisons between centres were not performed as this table provides descriptive context for interpreting external validation results rather than hypothesis testing.

Supplementary Table S1b. Demographic and Clinical Characteristics Comparison: Syphilis Dataset

| **Predictors** | **MSHC (N = 227995)** | **SSHC (N = 168443)** |
| --- | --- | --- |
| **Age, median (IQR)** | **29 (25-36)** | **30 (25-37)** |
| **Country of birth, n (%)** |  |  |
| Australia and New Zealand | 108755 (47.7) | 60336 (35.8) |
| Overseas | 108965 (47.8) | 108107 (64.2) |
| Missing | 10275 (4.5) | - |
| **Population type, n (%)** |  |  |
| MSM | 113152 (49.6) | 115933 (68.8) |
| Heterosexual male | 45683 (20) | 25516 (15.2) |
| Female | 69160 (30.3) | 26994 (16.0) |
| **Condom use with male partners, n (%)** | |  |
| Always | 42371 (18.6) | 43463 (25.8) |
| Sometimes | 95076 (41.7) | 79712 (47.3) |
| Never | 17584 (7.7) | 29221 (17.4) |
| Not Applicable | 3967 (1.7) | 11510 (6.8) |
| Unsure/Decline | 8966 (3.9) | 50 (0.0) |
| Missing | 60031 (26.3) | 4487 (2.7) |
| **Last time injected drugs not prescribed by a doctor, n (%)** | |  |
| Never | 213820 (93.8) | 165357 (98.2) |
| Less than 3 months | 3070 (1.3) | 1720 (1.0) |
| Within 3 -12 months | 1054 (0.5) | 709 (0.4) |
| More than 12 months | 2707 (1.2) | 657 (0.4) |
| Decline/Unsure | 5543 (2.4) | - |
| Missing | 1801 (0.8) | - |
| **Past history of gonorrhoea, n (%)** |  |  |
| Yes | 39599 (17.4) | 49445 (29.4) |
| No | 56334 (24.7) | 118982 (70.6) |
| Unsure | 3965 (1.7) | - |
| Missing | 128097 (56.2) | 16 (0.0) |
| **Past history of syphilis, n (%)** |  |  |
| Yes | 16152 (7.1) | 23550 (14.0) |
| No | 79781 (35.0) | 144877 (86.0) |
| Unsure | 3965 (1.7) | - |
| Missing | 128097 (56.2) | 16 (0.0) |
| **Infection positivity, n (%)** |  |  |
| Positive | 3894 (2.0) | 3410 (2.0) |
| Negative | 224101 (98.0) | 165033 (98.0) |

MSHC: Melbourne Sexual Health Centre; SSHC: Sydney Sexual Health Centre; MSM: men who have sex with men; IQR: interquartile range

Statistical comparisons between centres were not performed as this table provides descriptive context for interpreting external validation results rather than hypothesis testing.

Supplementary Table S1c. Demographic and Clinical Characteristics Comparison: Gonorrhoea Dataset

| **Predictors** | **MSHC (N = 262,599)** | **SSHC (N = 207582)** |
| --- | --- | --- |
| **Age, median (IQR)** | **29 (25-35)** | **29 (25-36)** |
| **Country of birth, n (%)** |  |  |
| Australia and New Zealand | 127958 (48.7) | 71583 (34.5) |
| Overseas | 122357 (46.6) | 135999 (65.5) |
| Missing | 12284 (4.7) | - |
| **Population type, n (%)** |  |  |
| MSM | 127500 (48.6) | 124963 (60.2) |
| Heterosexual Male | 40099 (15.3) | 37229 (18.0) |
| Female | 95000 (36.2) | 45390 (21.9) |
| **Condom use with male partners, n (%)** | |  |
| Always | 50959 (19.4) | 48936 (23.6) |
| Sometimes | 114329 (43.5) | 100084 (48.2) |
| Never | 23734 (9) | 38796 (18.7) |
| Not applicable | 4720 (1.8) | 13240 (6.4) |
| Unsure/Decline | 10218 (3.9) | 74 (0.0) |
| Missing | 58639 (22.3) | 6452 (3.1) |
| **Last time injected drugs not prescribed by a doctor, n (%)** | |  |
| Never | 245368 (93.4) | 204079 (98.3) |
| Less than 3 months | 3535 (1.3) | 1963 (1.0) |
| 3-12 months | 1211 (0.5) | 789 (0.4) |
| More than 12 months | 3134 (1.2) | 751 (0.4) |
| Decline/Unsure | 6366 (2.4) | - |
| Missing | 2985 (1.1) | - |
| **Past history of gonorrhoea, n (%)** |  |  |
| Yes | 46722 (17.8) | 61432 (29.6) |
| No | 67625 (25.8) | 146131 (70.4) |
| Unsure | 4696 (1.8) | - |
| Missing | 143556 (54.7) | 19 (0.0) |
| **Past history of syphilis, n (%)** |  |  |
| Yes | 17366 (6.6) | 28580 (13.8) |
| No | 96981 (36.9) | 178983 (86.2) |
| Unsure | 4696 (1.8) | - |
| Missing | 143556 (54.7) | 19 (0.0) |
| **Infection positivity, n (%)** |  |  |
| Positive | 15461 (5.9) | 15423 (7.4) |
| Negative | 247138 (94.1) | 192159 (92.6) |

MSHC: Melbourne Sexual Health Centre; SSHC: Sydney Sexual Health Centre; MSM: men who have sex with men; IQR: interquartile range

Statistical comparisons between centres were not performed as this table provides descriptive context for interpreting external validation results rather than hypothesis testing.

Supplementary Table S1d. Demographic and Clinical Characteristics Comparison: Chlamydia Dataset

| **Predictors** | **MSHC (N = 320355)** | **SSHC (N = 207582)** |
| --- | --- | --- |
| **Age, median (IQR)** | **29 (25-35)** | **29 (25-36)** |
| **Country of birth, n (%)** |  |  |
| Australia and New Zealand | 155603 (48.6) | 71583 (34.5) |
| Overseas | 149958 (46.8) | 135999 (65.5) |
| Missing | 14794 (4.6) | - |
| **Population type, n (%)** |  |  |
| MSM | 127410 (39.8) | 124964 (60.2) |
| Heterosexual male | 78703 (24.6) | 37228 (17.9) |
| Female | 114242 (35.7) | 45390 (21.9) |
| **Condom use with male partners, n (%)** | |  |
| Always | 53329 (16.6) | 48935 (23.6) |
| Sometimes | 127047 (39.7) | 100095 (48.2) |
| Never | 25462 (7.9) | 38795 (18.7) |
| Not Applicable | 4860 (1.5) | 13235 (6.4) |
| Unsure/Decline | 10640 (3.3) | 74 (0.0) |
| Missing | 99017 (30.9) | 6448 (3.1) |
| **Last time injected drugs not prescribed by a doctor, n (%)** | |  |
| Never | 301328 (94.1) | 204077 (98.3) |
| Less than 3 months | 3785 (1.2) | 1962 (1.0) |
| Within 3 -12 months | 1329 (0.4) | 789 (0.4) |
| More than 12 months | 3707 (1.2) | 754 (0.4) |
| Decline/Unsure | 6569 (2.1) | - |
| Missing | 3637 (1.1) | - |
| **Past history of gonorrhoea, n (%)** |  |  |
| Yes | 46193 (14.4) | 61420 (29.6) |
| No | 82874 (25.9) | 146143 (70.4) |
| Unsure | 5192 (1.6) | - |
| Missing | 186096 (58.1) | 19 (0.0) |
| **Past history of syphilis, n (%)** |  |  |
| Yes | 4092 (1.3) | 28557 (13.8) |
| No | 124975 (39.0) | 179006 (86.2) |
| Unsure | 5192 (1.6) | - |
| Missing | 186096 (58.1) | 19 (0.0) |
| **Infection positivity, n (%)** |  |  |
| Positive | 25870 (8.1) | 20801 (10.0) |
| Negative | 294485 (91.9) | 186781 (90.0) |

MSHC: Melbourne Sexual Health Centre; SSHC: Sydney Sexual Health Centre; MSM: men who have sex with men; IQR: interquartile range

Statistical comparisons between centres were not performed as this table provides descriptive context for interpreting external validation results rather than hypothesis testing.
